# Network Intrusion Detection Technology Based on Convolutional Neural Network and BiGRU

**DOI:** 10.1155/2022/1942847

**Published:** 2022-04-12

**Authors:** Bo Cao, Chenghai Li, Yafei Song, Xiaoshi Fan

**Affiliations:** School of Air and Missile Defense, Air Force Engineering University, Xi'an 710051, China

## Abstract

To solve the problem of low accuracy and high false-alarm rate of existing intrusion detection models for multiple classifications of intrusion behaviors, a network intrusion detection model incorporating convolutional neural network and bidirectional gated recurrent unit is proposed. To solve the problems of many dimensions of features and imbalance of positive and negative samples in the original traffic data, sampling processing is performed with the help of a hybrid sampling algorithm combining ADASYN and RENN, and feature selection is performed by combining random forest algorithm and Pearson correlation analysis; after that, spatial features are extracted by the convolutional neural network, and further features are extracted by incorporating average pooling and max pooling, and then BiGRU is used to extracts long-distance dependent information features to achieve comprehensive and effective feature learning. Finally, the Softmax function is used for classification. In this paper, the proposed model is evaluated on the UNSW_NB15, NSL-KDD, and CIC-IDS2017 data sets with an accuracy of 85.55%, 99.81%, and 99.70%, which is 1.25%, 0.59%, and 0.27% better than the same type model of CNN-GRU.

## 1. Introduction

Network intrusion detection is a security mechanism that has been developed in recent years to dynamically monitor, prevent, and defend against system intrusions. It mainly means that to find out whether the network system is attacked or violates the security policy by analyzing the information from several nodes of the network. Research on intrusion detection technologies at home and abroad has started since the 1980s and has now developed into an integral part of the network security architecture [1].

Traditional machine learning methods have been widely used in network intrusion detection systems, such as Bayesian [2–4], support vector machines [5–10], decision tree [11–13], logistic regression [14–16], and so on. They all have achieved good results. However, these methods are not suitable for massive and high-dimensional data, and they cannot improve their own sensitivity to outliers and noise, resulting in the degradation of classification performance. At the same time, due to the continuous development of digital technology, network attack methods are becoming more and more diversified, and the traditional machine learning methods have been difficult to meet the needs of users.

In recent years, deep learning techniques have been widely used in natural language processing [17], image recognition [18], and so on. It forms more abstract non-linear high-level representations by combining low-level features and then mines the input-output relationships between data, which has also achieved better results in the field of intrusion detection. Deep learning techniques commonly used in the field of intrusion detection include convolutional neural network (CNN), recurrent neural network (RNN), deep belief network, and so on. The literature converts the data traffic into individual pixel points in bytes to obtain the images generated by the traffic; then inputs the images into the convolutional neural network for convolution, pooling, and other operations; and finally obtains the classification results. The method achieves high accuracy in binary classification and multiclassification problems [19]. The literature uses the recognized KDD99 data set to conduct experiments, in which the long short-term memory (LSTM) network is used to complete the selection of parameters and achieve more satisfactory experimental results. However, the method leads to a high false-alarm rate due to the improper selection of training parameters [20]. A hierarchical intrusion detection system based on spatial and temporal features is proposed in the literature. It first learns low-level spatial features of network traffic by deep convolutional neural networks and then acquires high-level temporal features by LSTM, but the method does not consider the problems of feature fusion and data imbalance [21]. The paper combines the features of WaveNet and bidirectional gated recurrent unit (BiGRU) for feature extraction and proposes an intrusion detection method that fuses WaveNet and BiGRU. The model of the paper can achieve better detection accuracy but does not consider the problem of sample imbalance [22].

Now the intrusion detection techniques have made great progress, but there are also the following problems. First, it faces the problem of feature redundancy; more feature dimensions will not only increase the training time of the model but also reduce the detection effect of the model. An intrusion detection method based on principal component analysis (PCA) and recurrent neural network is proposed in the literature. The principal component analysis method is used to reduce the dimension and noise of the data to find out the principal component feature subset with the maximum information. Finally, the processed data is trained for classification using a recurrent neural network and achieves high accuracy [23]. The literature proposes an intrusion detection method by combining the advantages of an autoencoder and residual network. The feature extraction is performed by reconstructing the network with an autoencoder, and then the designed residual network is trained with the extracted features. The experimental results are better in terms of accuracy, true rate, and false-alarm rate [24]. Secondly, it faces the problem of unbalanced samples of positive and negative classes in the data set used to evaluate the effects of the model. The literature uses an improved local adaptive synthetic minority oversampling technique for unbalanced traffic data to achieve abnormal traffic detection using RNN that has high detection accuracy for different types [25].

In response to the above-mentioned problems, this paper designs an intrusion detection model incorporating CNN and BiGRU. Its main contributions are as follows:

1. For the problem of feature redundancy, this paper proposes a feature selection algorithm (RFP algorithm). It introduces the random forest algorithm to calculate feature importance and combines Pearson correlation analysis for feature selection.
2. For the problem of sample imbalance, this paper proposes a hybrid sampling algorithm (ADRDB algorithm) by combining the adaptive synthetic sampling (ADASYN) [26] and repeated edited nearest neighbors (RENN) [27] for sampling. At the same time, the density-based spatial clustering of applications with noise (DBSCAN) [28] is adopted to eliminate noise and finally obtain a balanced data set.
3. Spatial features are extracted by split-residual-fuse convolutional neural network (SRFCNN), and features with long-distance dependent information are extracted by BiGRU to fully consider the influence between the before and after attribute information to learn the data features comprehensively and effectively.

## 2. Related Work

Network security intrusion detection is a relatively broad area of research. Existing models used in the field of intrusion detection include convolutional neural networks, recurrent neural networks, machine learning, and hybrid models. Scholars have used a variety of different approaches to address the problems of low detection accuracy and difficulty in detecting a few classes of samples in the field of intrusion detection. Convolutional neural networks are mainly used in tasks related to image and video analysis, such as image classification, face recognition, target recognition, image processing, and so on. And, in recent years, it has also been widely used in the field of intrusion detection. A recurrent neural network is mainly used in various tasks of connected handwriting recognition and speech recognition. It is also widely used in the field of intrusion detection due to its effectiveness in processing time-series data.

In terms of improving detection accuracy, Tama et al. used a combination of particle swarm optimization algorithms, ant colony algorithms, and genetic algorithms for feature selection to reduce the feature size of the training data, followed by a secondary classification method to detect abnormal behavior in the network [29]. Bu and Cho combined a traditional learning classifier system with a convolutional neural network for the detection of anomalous behavior, and the proposed system has adaptive and learning capabilities [30]. Song et al. applied deep convolutional neural networks to intrusion detection systems, reducing the complexity of the models while also improving their detection accuracy [31]. Roy and Cheung proposed an IoT system based on a bidirectional long short-term memory recurrent neural network that achieves better results in detecting attacks [32]. Le et al. first performed feature selection via the SFSDT model, followed by classification via recurrent neural networks, achieving better results on both the NSL-KDD data set and the ISCX data set [33]. Hassan et al. proposed an intrusion detection system based on CNN and weight-dropped long short-term memory network and achieved more satisfactory results [34]. Tama and Lim used a parallel architecture to combine random forests, gradient boosters, and extreme gradient boosters to detect anomalous behavior with better results [35].

In terms of addressing the class imbalance: Louk et al. compared existing sampling methods and found that EasyEnsemble performed better in resolving sample imbalance [36]. Liu et al. divided the data set into hard and easy sets by ENN and reduced the imbalance of the original data set by processing the samples in the hard set through the K-means algorithm [37]. Yan et al. identified anomalous traffic with good accuracy by an improved density peak clustering algorithm [38]. Mulyanto M et al. introduced the focal loss function into the model to solve the problem of sample imbalance, with good results for both dichotomous and multiclassification problems [39]. Bedi P et al. propose an algorithm-level intrusion detection method that firstly fuses binary extreme gradient boosting (b-XGBoost), Siamese neural network (Siamese-NN), and deep neural network (DNN) for hierarchical filtration of input samples to identify attacks. And then it is classified by multiclass extreme gradient boosting classifier (m-XGBoost) [40].

## 3. The Network Intrusion Detection Model Incorporating CNN and BiGRU

Traditional intrusion detection models pay more attention to the features in time series and ignore spatial features in the process of detecting attacks. The use of a single convolutional neural network can lead to insufficient ability to extract features, which in turn results in low detection accuracy. The SRFCNN structure can extract the spatial features of data traffic more effectively and avoid the problem of gradient explosion while deepening the depth of the model. But its ability to extract long-distance dependent information is not good. BiGRU has a strong ability to extract long-distance dependency information; it can avoid the phenomenon of forgetting in the learning process, but its number of parameters is larger and the training time is longer. This paper integrates the two models to improve the ability to learn features, which can fully extract features from both spatial and temporal dimensions, and then achieve higher classification detection accuracy.

The proposed network intrusion detection model integrating convolutional neural network and BiGRU consists of three main stages.

First, preprocessing stage. Convert the original traffic data into numerical features and normalize them, balance the data set by hybrid sampling method, and finally extract features by RFP algorithm.

Second, training stage. The preprocessed data were extracted by SRFCNN network and BiGRU and finally classified by Softmax classifier.

Third, testing phase. Pass the test set to the trained model for classification.

**Figure 1:**
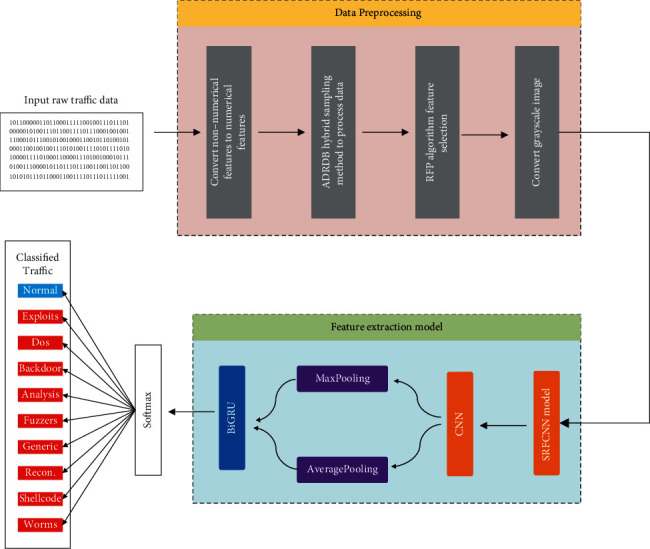
Network intrusion detection model incorporating SRFCNN and BiGRU.

The structure diagram of the proposed model in this paper is shown in Figure 1.

### 3.1. Data Preprocessing

**Figure 2:**
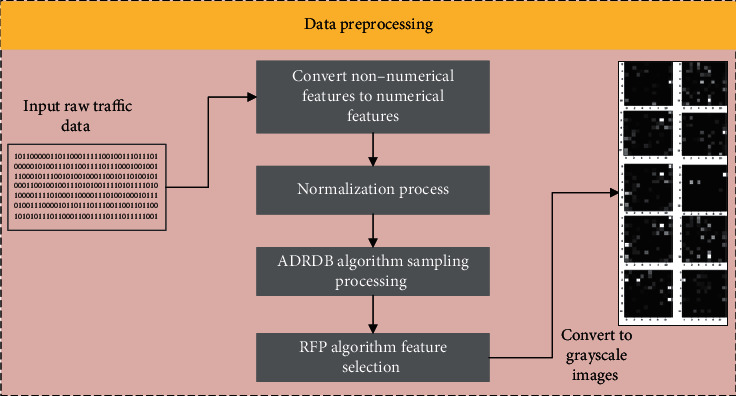
Preprocessing traffic chart.

In the preprocessing stage, this paper firstly converts the non-numerical features in the original traffic data into numerical features and normalizes the features; secondly, a hybrid sampling algorithm (ADRDB algorithm) combining ADASYN and RENN is used for sampling; afterwards, feature selection is performed by the feature selection algorithm (RFP algorithm); finally, the obtained data is converted into grayscale maps. The specific process of this stage is shown in Figure 2.

#### 3.1.1. Non-Numerical Feature Transformation and Normalization

The only way the traffic data can be used as model input is after cleaning, labeling, annotation, and preparation. In this paper, the LabelEncoder function in scikit-learn is used to convert the non-numeric features in the raw data traffic to numeric features to ensure that all data are numeric, so as to facilitate the model to learn the data features.

After the traffic features are converted to numeric, it is easy to ensure that the clustering of sample points in the feature space will be guided by individual feature values and less influenced by other feature values due to the different sizes of the taken values. Data normalization can reduce the variance of the features to a certain range and reduce the influence of outliers. In this paper, we use min-max normalization to normalize the feature values to between 0 and 1, as shown in the following formula:

(1) $$\begin{array}{c} h_{i,j}=\frac{h_{i,j}-\text{min}\left(h_{i,j}\right)}{\max\left(h_{i,j}\right)-\min\left(h_{i,j}\right)}, \end{array}$$

where *h*_*i*,*j*_ represents the feature value of row *i* and column *j* in the data set.

After the values are normalized, the majority class and minority class samples are balanced by the proposed hybrid sampling algorithm to obtain the balanced data set. After that, the useful features are extracted by the feature selection algorithm.

#### 3.1.2. Hybrid Sampling Method Combining ADASYN and RENN

The core idea of the hybrid sampling method combining ADASYN and RENN is mainly divided into the following sections: firstly, the original data set is divided into majority and minority sample sets. The new majority sample set is obtained by undersampling through the RENN algorithm, and the new minority sample set is obtained by oversampling with the ADASYN algorithm. Afterwards, the new data set obtained by merging the two is passed through the DBSCAN clustering algorithm to remove the noise and obtain the balanced data set. The hybrid sampling method combining ADASYN and RENN is specified as follows.

The inputs of the algorithm are the original majority sample set N and minority sample set P and the number of samples. The outputs are the balanced majority sample set newN and minority sample set newP (Algorithm 1).

1. Calculate the imbalanced degree of the data set.
2. If *d* < *d*_*th*_ (where *d*_*th*_ is the predetermined value of the maximum allowed degree of imbalance ratio), perform the following operations: firstly, calculate the number of samples that need to be generated for the minority class; secondly, for each sample in N, find its *k*_1_ nearest neighbors and calculate the ratio *r*_*i*_, where Δ_*i*_ denotes the number of samples *x*_*i*_ belonging to the majority class among the *k* nearest neighbors. |X| represents the number of samples; after that, normalize *r*_*i*_ to ${\hat{r}}_{i}$; finally, calculate the number of samples that need to be synthesized for each minority class sample.
3. For each sample in N, *g*_*i*_ samples are obtained according to the steps to obtain a new minority sample set.
4. For each sample in P, select *k*_2_ nearest neighbor from newN.
5. Calculate the number of minority samples in the nearest neighbors of each majority sample and reject the sample if the number of samples is greater than *e*. In this paper, *e* = 1.
6. Repeat (4) and (5) to generate a new set of majority samples.
7. Eliminate the noise in newP and newN to get the final newN and newP.

#### 3.1.3. Feature Selection Algorithm

To address the problem of feature redundancy in data sets, this paper proposes a new feature selection algorithm. The algorithm first calculates the importance degree of each feature of the sample by the random forest algorithm and ranks them according to the importance degree; after that, it calculates the correlation between features by Pearson correlation coefficient; finally, it combines the two obtained results to achieve feature selection.

Random forest algorithm (RF) is an ensemble learning algorithm based on the decision tree. In feature engineering, the RF algorithm can identify important features from a large number of sample features; its essence is to analytically calculate the contribution of each feature of the sample on the tree and later calculate its average and compare the magnitude of the contribution between features to identify the important features [41]. Existing methods are usually evaluated using the Gini index or the out-of-bag data error rate as evaluation metrics; the specific steps are as follows:

(1) For each base learner, select the corresponding out-of-bag data to calculate its error, denoted as error_a.
(2) Randomly add disturbances to all samples of out-of-bag data and calculate its error, denoted as error_b.
(3) Assuming that the forest contains *M* trees, the importance value of a feature can be calculated by the following equation:
  (2) $$\begin{array}{c} \text{Importance}=\frac{\text{error}\_b-\text{error}\_a}{M}. \end{array}$$
(4) Filter out the features with higher importance to construct a new data set.

Pearson correlation coefficient is used to measure the correlation between two variables *X* and Y, which takes values in the range (–1, 1) [42]. The Pearson correlation coefficient between the two features is obtained by calculating the covariance and standard deviation between the two eigenvalues and quotienting them with the following formula:

(3) $$\begin{array}{c} \rho_{X,Y}=\frac{\mathrm{cov}\left(X,Y\right)}{\sigma_{X}\sigma_{Y}}=\frac{E\left[\left(X-\mu_{X}\right)\left(Y-\mu_{Y}\right)\right]}{\sigma_{X}\sigma_{Y}}. \end{array}$$

The Pearson correlation coefficient varies from −1 to 1. If the Pearson correlation coefficient of two characteristics is close to ±1, it indicates a high correlation between them, and the relationship between them can be well expressed by a linear equation. If the Pearson correlation coefficient is close to 0, it means that there is no linear relationship between the two features. The pseudo-code of the feature selection algorithm proposed in this paper is shown in Algorithm 2.

**Figure 3:**
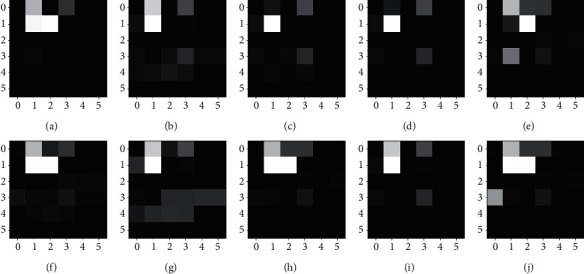
Converted grayscale map: (a) Normal, (b) Analysis, (c) Backdoor, (d) Dos, (e) Exploits, (f) Fuzzers, (g) Generic, (h) Reconnaissance, (i) Shellcode, and (j) Worms.

The raw traffic data are converted into grayscale maps after feature selection. The converted grayscale plots for different categories are shown in Figure 3.

### 3.2. Model Structure

One of the main advantages of CNN over traditional classification methods is that it attempts to learn the best filters on its own. The existing popular CNN structures mainly include residual network (ResNet) [43] and inception network [44]. ResNet proposes a concept of split-transform-merge.

**Figure 4:**
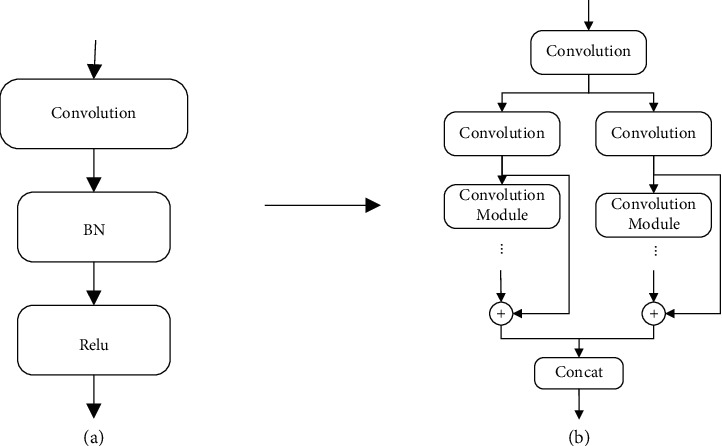
SRFCNN model diagram: (a) convolution module and (b) SRFCNN structure.

In order to improve the expressiveness of CNN and to fully learn the diversity of features in the classification process, a new convolution neural network based on separation-residual-fusion is proposed in this paper according to the relevant ideas of the residual neural network, and the specific structure is shown in Figure 4. After the data is input, it is split into different paths by the segmented block convolutional neural network, and then different types of residual transformation are carried out for each segmented feature. As shown in the figure, the layers of each residual are different, so as to ensure that it can learn simple to complex feature transformation. Finally, the feature maps after the residual neural network are fused. The application of the residual network can effectively solve the gradient explosion problem caused by the increased depth of the network. Two-dimensional convolution has shown excellent performance in the field of computer vision, so this paper uses 2D convolution to extract the spatial features of the data.

**Figure 5:**
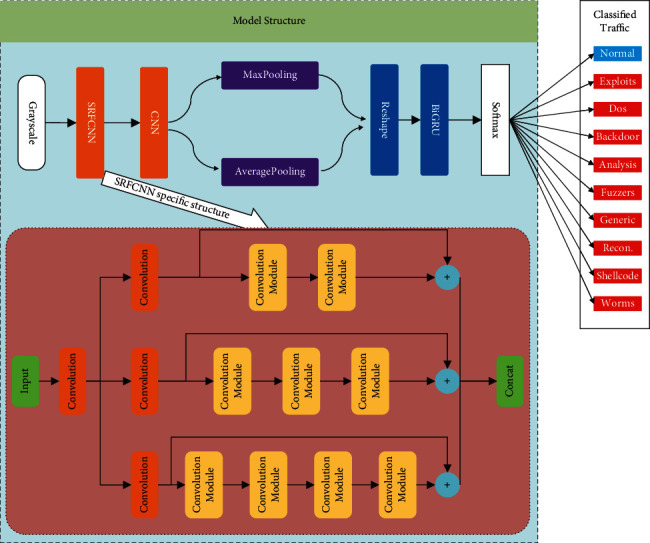
Model structure diagram.

The intrusion detection model proposed in this paper consists of three main parts: in order to comprehensively and finitely learn the features of the data, firstly, the spatial features of the data are extracted by SRFCNN; secondly, the feature extraction capability is further enhanced by fusing average pooling and max pooling; afterwards, the temporal features are extracted by BiGRU, and finally, the classification is carried out by Softmax. The specific structure of the model is shown in Figure 5:

1. The grayscale map obtained after preprocessing is input to the SRFCNN network to extract spatial features and obtain the output *F*
2. The new feature map *F* is aggregated with spatial information by fusing max pooling and average pooling to obtain the new feature map *F*_*C*_
3. Pass *F*_*C*_ into the BiGRU unit to extract the dependencies between features and obtain the output *F*_*G*_
4. Pass *F*_*G*_ into the fully connected layer that uses Softmax as the activation function to achieve the classification of intrusion detection behavior

## 4. Experimental Results and Analysis

### 4.1. Experimental Setup

In order to test the performance of the proposed network intrusion detection method combining CNN and BiGRU, this paper designs multiple sets of experiments.

  Experiment 1: Feature selection analysis experiment
  Experiment 2: Experiment on the number of SRFCNN modules
  Experiment 3: Comparison experiment between single model and hybrid model
  Experiment 4: Comparison experiment of different feature selection methods
  Experiment 5: Comparison experiment of different sampling methods
  Experiment 6: Comparison experiment of different pooling methods
  Experiment 7: Performance analysis and comparison experiment

The intrusion detection model experiments and comparison experiments proposed in this paper are conducted on a 64 bit Windows Intel® Core™ i7-7700HQ CPU (2.80 GHz) with 16 GB RAM and a python-based Nvidia GeForce GTX 1050 GPU (4 GB), using Python's TensorFlow library to write the SRFCNN and BiGRU models for this paper.

### 4.2. Data Set and Evaluation Criteria

Over the years, many data sets related to intrusion detection have been introduced for research and development, including KDDCup99 [45], UNSW-NB15 [46], NSL-KDD [47], and CIC-IDS2017 [48]. In this paper, we choose to use the UNSW-NB15, NSL-KDD, and CIC-IDS2017 data sets to evaluate the proposed model.

The NSL-KDD data set is an improvement of the KDD99 data set, which removes the redundant and duplicate data from the training and test sets on the basis of the KDD99 data set so that the training and test sets are set up in a more reasonable way. It mainly contains 41-dimensional attribute features and 1-dimensional category features, covering 5 types of Normal, Probe, Dos, R2L, and U2R. The number of samples of different categories in the NSL-KDD data set is shown in Table 1.

The UNSW-NB15 data set is a new data set generated in 2015 by the Cyber Range Laboratory of the Australian Centre for Cyber Security (ACCS) using the IXIA PerfectStorm tool to simulate realistic cyber environments. The data set mainly consists of 47 attribute features and 2 category features and contains 9 types of attacks: Fuzzers, Analysis, Backdoors, DoS, Exploits, Generic, Reconnaissance, Shellcode, and Worms. This paper directly uses the partitioned training set and testing set to test the performance of the model. The number of samples of different categories in the UNSW_NB15 data set is shown in Table 2.

The CIC-IDS2017 data set is derived from the July 3–7, 2017 Canadian Institute for Cybersecurity (CIC) collection for cyber data, which contains benign as well as recent common attacks in the field of cyber intrusions, filling the gap of no cyber-based attacks in the UNSW-NB15 data set. The data set contains 78-dimensions of attribute features and 1-dimension of category features covering 15 attack types. In this paper, the anomalous behaviors of similar nature are merged, and the final data set contains 10 types of attacks: BENING, Dos, Portscan, Ddos, Patator, Bot, Web attack, Infiltration, and Heartbleed. The number of samples of different categories in the CIC-IDS2017 data set is shown in Table 3.

The evaluation metrics of the network security intrusion detection model include four main metrics: precision, accuracy, recall, and F1-score. In the specific detection results, T (true) and F (false) represent correctly or incorrectly classified data, respectively. P (positive) and N (negative) indicate that the predicted results of the detection system are abnormal or normal data, respectively. All data in the data set must be classified into four categories: TP, TN, FP, and FN. Only TP indicates that the system classification result consists of abnormal attack data with correct classification result; TN indicates that the system classification result is positive and correct; FP indicates that the system predicts the data as abnormal attack data, but the classification result is wrong; and FN indicates that the system predicts the data as normal data, but the classification result is incorrect. The classification results of the model for the data are represented by the confusion matrix, as shown in Table 4.

The accuracy describes the ratio of the number of correctly predicted samples to the total sample number and is calculated as follows:

(4) $$\begin{array}{c} \text{Accuracy}=\frac{TP+TN}{TP+FP+TN+FN}. \end{array}$$

The precision describes the ratio of the number of classes predicted to be positive to the number of classes actually predicted to be positive and is calculated as follows:

(5) $$\begin{array}{c} \text{precision}=\frac{TP}{TP+FP}. \end{array}$$

The recall describes the ratio of the number of predicted positive classes that are actually positive to the number of all positive classes and is calculated as follows:

(6) $$\begin{array}{c} \text{recall}=\frac{TP}{TP+FN}. \end{array}$$

The *F*_1_ − score describes the magnitude of the harmonic mean between precision and recall, calculated as follows:

(7) $$\begin{array}{c} F_{1}-\text{score}=\frac{2\times \text{recall}\times \text{precision}}{\text{recall}+\text{precision}}. \end{array}$$

It can be seen that F1 achieves larger values when both recall and precision have larger values.

### 4.3. Experimental Simulation

In order to fully verify the effectiveness of the model proposed in this paper, multiple sets of experiments are set up in this paper: Section 4.3.1 sets up feature selection analysis experiments to introduce the process and results of feature selection in detail.  Section 4.3.2 sets up comparison experiments of SRFCNN module numbers to dynamically adjust the number of modules through experiments.  Section 4.3.4 compares different feature selection methods to compare the advantages and disadvantages of the proposed RFP algorithm compared with existing algorithms used in intrusion detection.  Section 4.3.5 compares different sampling methods to compare the advantages and disadvantages of the proposed ADRDB algorithm compared with other sampling methods.  Section 4.3.6 compares different pooling methods to analyze the effects of using one pooling method alone and mixed pooling on the performance of the model.  Section 4.3.7 set up performance analysis and comparison experiments to analyze the convergence of the model and compare it with existing models.

#### 4.3.1. Feature Selection Analysis Experiment

**Figure 6:**
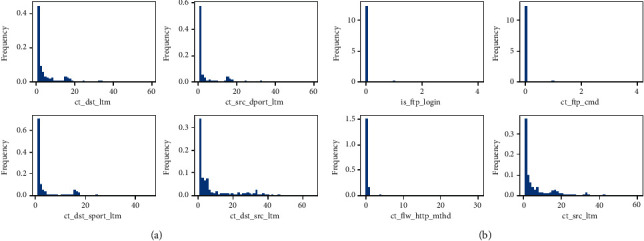
Histogram of feature distribution of UNSW_NB15 data set.

**Figure 7:**
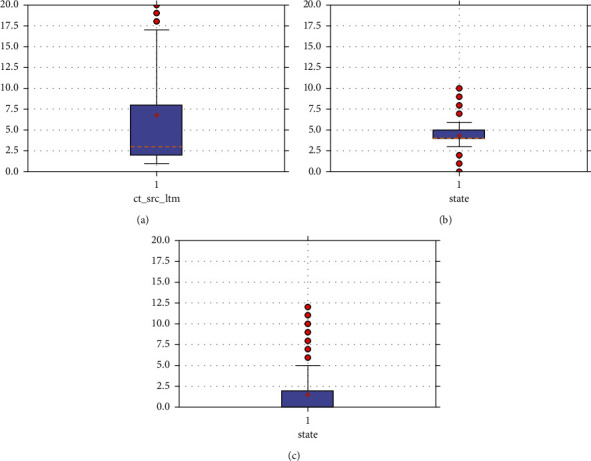
Box plot of feature distribution of UNSW_NB15 data set.

In order to verify the classification performance of the CNN-GRU algorithm proposed in this paper, the public data sets UNSW_NB15, NSL-KDD, and CIC-IDS2017 were selected. This section focuses on the UNSW_NB15 data set for the detailed introduction. In order to visualize the distribution of each feature, it was demonstrated by histograms and box plots. The histograms and box line plots of some features are shown in Figures 6 and 7.

Since some features do not have outliers, Figure 7 shows only a few features with outliers. The analysis shows that there are a few outliers in the six types of features: spkts, dpkts, ct_src_ltm, ct_srv_dst, ct_srv_src, and ct_state_ttl, which have a small impact on the whole data set. However, state, dur, sloss, dloss, service, ct_dst_ltm, ct_src_dport_ltm, st_dst_sport_ltm, tcprtt, synack, and ackdat have more outliers.

**Figure 8:**
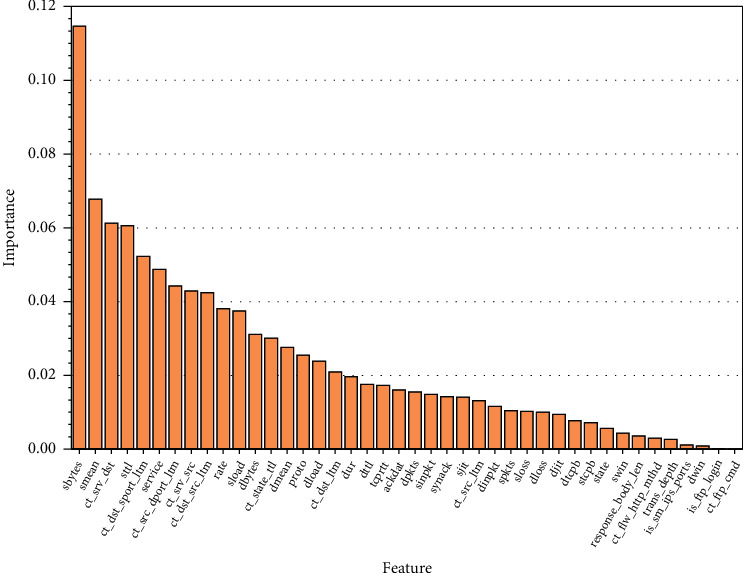
Feature importance graph of UNSW_NB15 data set.

In this paper, the importance of each feature in the data set UNSW_NB15 is first calculated by the random forest algorithm and ranked according to the degree of importance, as shown in Figure 8. It can be seen from the figure that the importance degree of different features varies widely, such as the importance value of feature sbytes is 0.115, while the importance values of is_ftp_login and ct_ftp_cmd are 0. The importance metrics of all features are distributed between 0 and 0.12.

**Figure 9:**
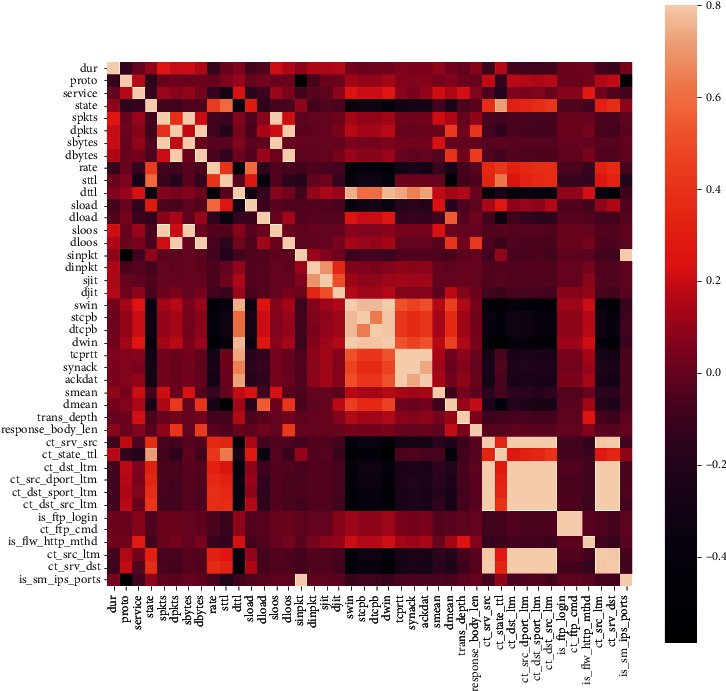
Heat map of UNSW_NB15 data set features.

**Figure 10:**
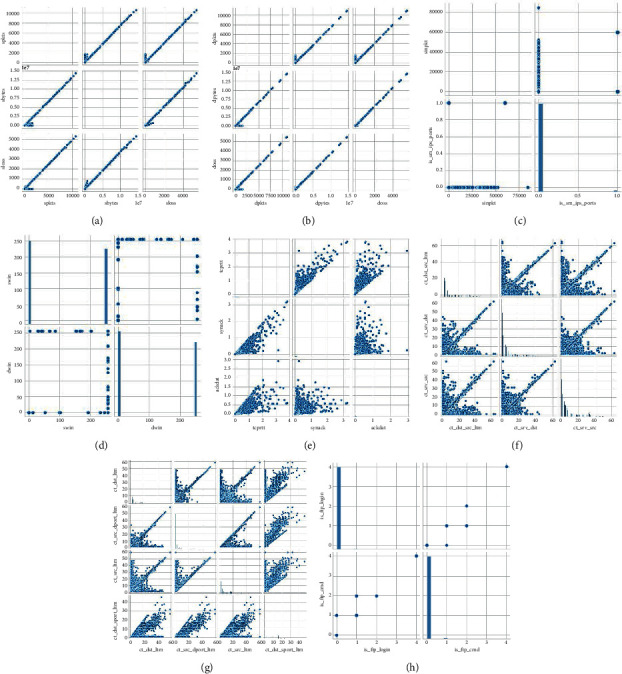
Correlation plot between features of UNSW_NB15 data set. (a) spkts, sbytes, and sloss; (b) dpkts, dbytes, and dloss; (c) sinpkt and is_sm_ips_ports; (d) swin and dwin; (e) tcprtt, synack, and actdat; (f) ct_dst_src_ltm, ct_srv_dst, and ct_srv_src; (g) ct_dst_ltm, ct_src_dport_ltm, ct_src_ltm, and ct_dst_sport_ltm; and (h) is_ftp_login and ct_ftp_cmd.

Feature selection based only on feature importance is a single reference criterion, and the results obtained are not very convincing, so this paper combines feature importance and Pearson correlation analysis for feature selection. In order to visualize the correlation between features, a feature correlation diagram is established as shown in Figure 9. The correlation between these 42 features can be clearly seen from the figure. And the lighter and darker parts in the figure clearly show the strong correlation between the two types of features. To further observe whether features *X* and *Y* present correlation in the plane distribution, a correlation graph with feature *X* as the *x*-axis and feature *Y* as the *y*-axis is established. Because of the large number of data feature dimensions, this paper selects the cases where the correlation index of the two types of features is greater than or equal to 0.9 or less than or equal to –0.9 for analysis and introduction, as shown in Table 5, and the correlation graph established between features *X* and *Y* is shown in Figure 10.

From Figure 10(a), we can see that spkts, sbytes, and sloss show linear correlation. Figure 10(b) shows that dpkts, dbytes, and dloss show linear correlation. From Figure 10(c), we can see that sinpkt and is_sm_ips_ports are not linearly correlated. Figure 10(d) shows that the two types of features, swin and dwin, are linearly uncorrelated, and their multiple values are only 0 and 255. Figure 10(e) shows that there are some similarities between “tcprtt” and “synack” as well as “tcprtt” and “actdat.” As the value of *x* increases, the value of *y* also increases, but the values of synack and ackdat are relatively scattered. From Figure 10(f), we can see that the values of ct_dst_src_ltm, ct_srv_dst, and ct_srv_src features are relatively dispersed, but there are still some linear relationships. Figure 10(g) shows that the values of ct_dst_ltm, ct_src_dport_ltm, ct_src_ltm, and ct_dst_sport_ltm are relatively scattered, and there are also some linear relationships. Figure 10(h) shows that the values of is_ftp_login and ct_ftp_cmd are linearly unrelated.

Combining Figures 8 and10 for feature selection, for features with strong linear correlation, the more important features are retained according to the importance degree; for features with weak linear correlation, the importance index of the features is analyzed, and if they are lower than 0.001, they are eliminated; for features whose correlation index is not within the analysis interval, their importance index is also analyzed, and features with importance degree lower than 0.0001 are eliminated. Finally, the NSL-KDD data set leaves 28-dimensional features; the UNSW_NB15 data set leaves 28-dimensional features; and the CIC-IDS2017 data set leaves 52 features.

#### 4.3.2. Experiment on the Number of SRFCNN Modules

In order to select the best number of SRFCNN modules, this section sets a comparison experiment with different numbers of modules: under the same experimental conditions, the grayscale maps obtained after preprocessing are input to the SRFCNN with the number of modules 2, 3, 4 and 5 to extract features and test them, and the classification accuracy, precision, and F1-score values are shown in Table 6.

It is experimentally demonstrated that for the NSL-KDD data set, the results obtained with five modules are better with the accuracy, precision, and F1-score of 99.81%, 99.76%, and 99.79%, respectively. For the UNSW_NB15 data set, the results obtained with three modules are better with the accuracy, precision, and F1-score of 85.55%, 86.24%, and 85.61%, respectively. For the CIC-IDS2017 data set, the results obtained with three modules were better, with the accuracy, precision, and F1-score of 99.70%, 99.68%, and 99.69%, respectively. Therefore, this paper uses SRFCNN fused with five residual modules to extract spatial features of the NSL-KDD data set, SRFCNN fused with three residual modules to extract spatial features of the UNSW_NB15 data set, and SRFCNN fused with three residual modules to extract spatial features of the CIC-IDS2017 data set.

#### 4.3.3. Comparison Experiment between Single Model and Hybrid Model

To verify the effectiveness of the model proposed in this paper on intrusion recognition, this section sets performance analysis experiments on the intrusion detection model combining SRFCNN and BiGRU: SRFCNN, BIGRU, and hybrid models are tested by the NSL-KDD, UNSW-NB15, and CIC-IDS2017 data sets under the same experimental conditions, and their classification accuracy, precision, recall, and F1-score values are obtained as shown in Table 7.

It can be seen from Table 7 that compared with using the single model of SRFCNN and BiGRU, the hybrid model combining SRFCNN and BIGRU can effectively extract the features of the raw data traffic and then effectively achieve intrusion detection. The detection accuracy, recall, precision, and F1 score of data set NSL-KDD reached 99.81%, 99.81%, 99.76%, and 99.79%, respectively; the detection accuracy, recall, precision, and F1 score of data set UNSW_NB15 reached 85.55%, 85.55%, 86.24%, and 85.61%, respectively; and the detection accuracy, recall, precision, and F1 score of data set CIC-IDS2017 reached 99.70%, 99.70%, 99.68%, and 99.69%, respectively. The reason is that SRFCNN can learn spatial features effectively by deepening the depth and width of the network, while BiGRU can extract temporal features of the data better. The model in this paper combines SRFCNN and BiGRU to learn both spatial and temporal features of the data to achieve effective and comprehensive learning of the features, thus achieving better results.

#### 4.3.4. Comparison Experiments of Different Feature Selection Methods

In order to verify the effectiveness and applicability of the feature selection method proposed in this paper, a comparison experiment of different feature selection methods is set up in this section: the feature selection method (RFP) proposed in this paper is compared with existing feature selection methods such as PCA [23] and AE [24] under the same experimental conditions. The features of the NSL-KDD data set are reduced to 28 dimensions; the features of the UNSW_NB15 data set are reduced to 28 dimensions; and the features of the CIC-IDS2017 data set are reduced to 52 dimensions by the above 3 methods. The results are shown in Table 8.

From Table 8, it can be seen that the data processed by the RFP algorithm proposed in this paper are used in the model can achieve better results. It is found that PCA relies more on variance when performing data dimensionality reduction, but the non-principal components with small variance may also contain important information on sample differences, and the dimensionality reduction process will have an impact on the subsequent data processing. AE relies more on the training data when performing feature space reconstruction. So both methods do not achieve better results. The RFP algorithm proposed in this paper starts from the data itself and selects features according to their importance degree and relevance to achieve the effect of improving the classification accuracy of the model.

#### 4.3.5. Comparison Experiments of Different Sampling Methods

In order to solve the problem of the unbalanced data set, this paper adopts the sampling methods of mixed ADASYN and RENN to process the data set. In order to verify the effectiveness of the proposed method, this section sets the comparison experiment of different sampling methods: under the same experimental conditions, the model adopts SMOTE, ADASYN, random undersampling, random oversampling, ENN, RENN, and ADRDB to process the imbalance data set. The detection results are shown in Table 9.

From Table 9, it can be seen that comparing many different sampling methods, the ADRDB proposed in this paper, which integrates ADASYN and RENN, has a better treatment effect for sample imbalance. The reasons are that the single oversampling methods such as random oversampling, SMOTE, and ADASYN cannot effectively discriminate the noisy data and easily generate a large amount of noisy data in the process of synthesizing new samples, which leads to the degradation of the model classification effect; the single undersampling methods such as random undersampling, ENN, and RENN easily tend to lose the key information of most classes of samples, resulting in lower classification results. The ADRDB samples the majority and minority samples separately and rejects the noisy data by the DBSCAN algorithm, which not only avoids the loss of key information but also reduces the influence of noisy data on the classifier model, thus achieving better results.

#### 4.3.6. Comparison Experiments of Different Pooling Methods

In this paper, we adopt fusion max pooling and average pooling to solve the problem of insufficient feature extraction ability of the model. To verify the effectiveness of the proposed method, this section sets comparison experiments of different pooling methods: under the same experimental conditions, the model adopts three different methods of average pooling, max pooling, and fusion pooling to extract features. The detection accuracy is shown in Table 10.

From Table 10, it can be seen that the fusion pooling method is more effective. The reason is that the average pooling is used to extract features by averaging the global range of features to achieve feature learning, while the max pooling is used to extract features by taking the maximum value of the feature points in the domain, and the fusion of the two pooling methods can make up for each other and fully learn the features. The experimental results show that fusion pooling effectively improves the model's ability to learn features, and the classification results are greatly improved.

#### 4.3.7. Performance Analysis and Comparison Experiments

**Figure 11:**
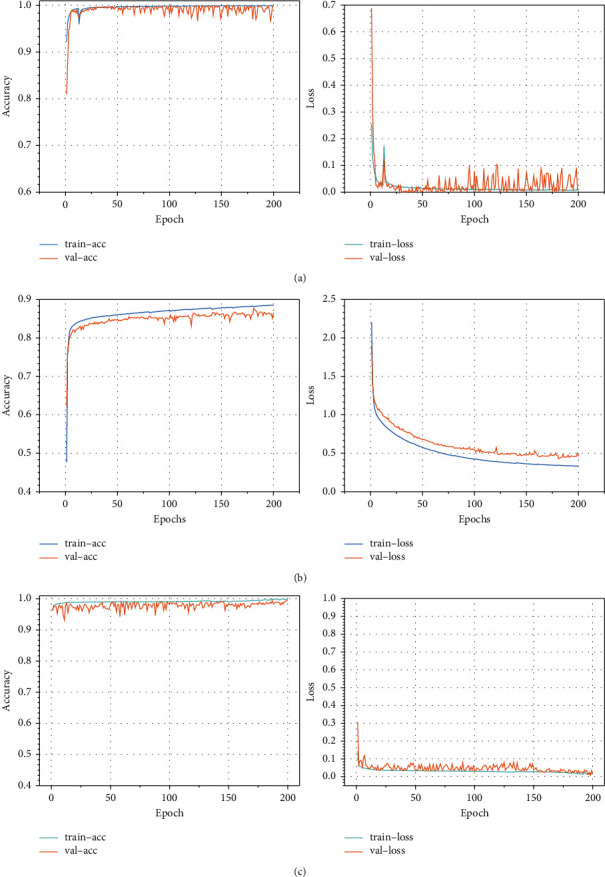
Accuracy and loss value with the number of iteration steps curve: (a) accuracy and loss value with the number of iteration steps curve of the NSL-KDD data set; (b) accuracy and loss value with the number of iteration steps curve of the UNSW_NB15data set; (c) accuracy and loss value with the number of iteration steps curve of the CIC-IDS2017 data set.

Figure 11 gives the classification result accuracy and loss value variation curves with the number of iteration steps for the intrusion detection model combining SRFCNN and BiGRU. From Figure 11, it can be seen that the model in this paper achieves a better convergence effect.

To further verify the effectiveness of the intrusion detection model proposed in this paper, this section sets performance comparison experiments: under the same experimental conditions, common machine learning methods such as random forest, K-means clustering, decision tree, and the recently proposed intrusion detection model are applied to the data set. The performance comparison is shown in Table 11.

From Table 11, it can be seen the proposed model achieves better results in all evaluation indexes. The reasons are that compared with machine learning algorithms, the model in this paper learns features through neural networks, which can form a more abstract and non-linear high-level representation by combining low-level features and then exploit the input-output relationship between data, which effectively improves the accuracy of intrusion detection. Compared with S-ResNet, CNN, CNN-GRU, CNN-LSTM, and CNN-BiLSTM models, the intrusion detection model incorporating SRFCNN and BiGRU extracts and learns both spatial and temporal features of the data, and the extracted feature information is more comprehensive, thus achieving better results.

## 5. Conclusions

To solve the problems of incomplete feature extraction and the general multiclassification effect of general intrusion detection models, this paper proposes an intrusion detection model fusing convolutional neural network and bidirectional gated recurrent unit. The model solves the problems of the unbalanced data set and feature redundancy by ADRDB and RFP algorithm and then achieves comprehensive and sufficient learning of features by fusing SRFCNN and BiGRU. Finally, feature selection analysis experiments, hybrid model versus single model comparison experiments, feature extraction method comparison experiments, pooling method comparison experiments, and performance analysis experiments on the data set prove that the model has strong feature extraction capability, high detection accuracy, and low false-alarm rate when processing large-scale and high-dimensional network data, providing some research support for intrusion detection systems.

## Figures and Tables

**Algorithm 1:**
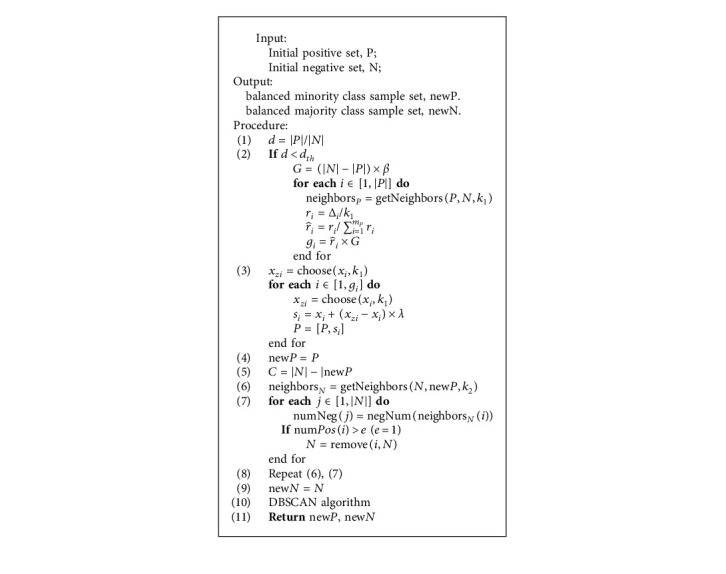
Hybrid sampling method incorporating ADASYN and RENN (ADRDB).

**Algorithm 2:**
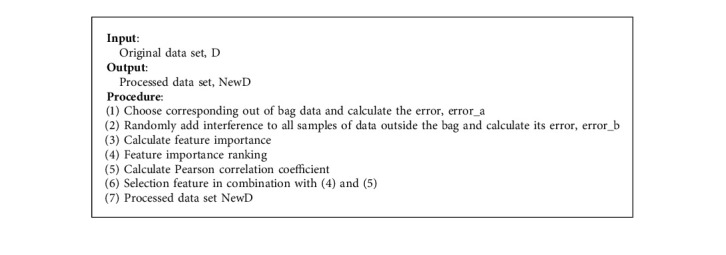
Feature selection algorithm (RFP).

**Table 1:** Distribution of different attack behaviors in the NSL-KDD data set.

Data set	Attack behavior					Total
	Normal	Dos	Probe	R2L	U2R	
KDDTrain+	67,343	45,927	11,656	995	52	125,973
KDDTest+	9,889	7,460	2,707	2,421	67	22,544
Total	77,232	53,387	14,363	3,416	119	148,517

**Table 2:** Distribution of different attack behaviors in the UNSW-NB15 data set.

Data set	Attack behavior										Total
	Normal	Fuzzers	Analysis	Backdoors	DoS	Exploits	Generic	Recon.	Shellcode	Worms	
Train	56,000	18,174	2,000	1,746	12,264	33,393	40,000	10,491	1,133	130	175,341
Test	37,000	6,062	677	583	4,089	11,132	18,871	3,496	378	44	82,332
Total	93,000	24,246	2,677	2,329	16,353	44,525	58,871	13,987	1,511	174	257,673

**Table 3:** Distribution of different attack behaviors in the CIC-IDS2017 data set.

Data set	Attack behavior									Total
	Benign	Dos	PortScan	Patator	Ddos	Bot	Web attack	Infiltration	Heartbleed	
Train	1,654,737	176,863	111,251	9,685	89,619	2,752	1,526	25	7	2,046,465
Test	709,173	75,798	47,679	4,150	38,408	1,180	654	11	4	877,057
Total	2,363,910	252,661	158,930	13,835	128,027	3,932	2,180	36	11	2,923,522

**Table 4:** Confusion matrix.

Classification	Predicted positive category	Prediction negative category
Actual positive category	TP	FN
Actual negative category	FP	TN

**Table 5:** Feature correlation index.

Feature		Correlation index
Spkts	sbytes	0.964
Spkts	sloss	0.972
dpkts	dbytes	0.973
dpkts	dloss	0.980
sbytes	sloss	0.996
dbytes	dloss	0.997
sinpkt	is_sm_ips_ports	0.942
swin	dwin	0.980
ct_dst_src_ltm	ct_srv_dst	0.960
tcprtt	synack	0.943
tcprtt	ackdat	0.920
ct_srv_src	ct_dst_src_ltm	0.954
ct_srv_src	ct_srv_dst	0.949
ct_dst_ltm	ct_src_dport_ltm	0.962
ct_dst_ltm	ct_src_ltm	0.902
ct_src_dport_ltm	ct_dst_sport_ltm	0.908
ct_src_dport_ltm	ct_src_ltm	0.909
is_ftp_login	ct_ftp_cmd	0.999

**Table 6:** Comparison of the results of the number of modules.

Data set	Number of modules	Accuracy	Precision	F1
NSL-KDD	2	0.967	0.977	0.971
	3	0.985	0.987	0.986
	4	0.987	0.987	0.987
	**5**	**0.998**	**0.998**	**0.998**
UNSW_NB15	2	0.843	0.840	0.841
	**3**	**0.856**	**0.862**	**0.856**
	4	0.841	0.838	0.839
	5	0.823	0.822	0.804
CIC-IDS2017	2	0.984	0.985	0.984
	**3**	**0.997**	**0.997**	**0.997**
	4	0.980	0.982	0.980
	5	0.981	0.983	0.981

**Table 7:** Comparison of single model and hybrid model.

Data set	Module	Evaluation indicators (%)			
		Accuracy	Precision	Recall	F1-score
NSL-KDD	SRFCNN	98.65	98.72	98.65	98.67
	BiGRU	98.97	99.17	98.97	99.07
	SRFCNN-BiGRU	**99.81**	**99.76**	**99.81**	**99.79**
UNSW_NB15	SRFCNN	84.01	88.62	84.01	85.35
	BiGRU	82.58	84.73	82.58	83.64
	SRFCNN-BiGRU	**85.55**	**86.24**	**85.55**	**85.61**
CIC-IDS2017	SRFCNN	93.75	96.07	93.75	94.56
	BiGRU	98.40	98.59	98.40	98.41
	SRFCNN-BiGRU	**99.70**	**99.68**	**99.70**	**99.69**

**Table 8:** Comparison of different feature selection methods.

Data set	Feature selection method	Evaluation indicators (%)			
		Accuracy	Precision	Recall	F1-score
NSL-KDD	RFP	**99.81**	**99.76**	**99.81**	**99.79**
	PCA	98.74	98.87	98.74	98.79
	AE	98.70	98.81	98.70	98.81
UNSW_NB15	RFP	**85.55**	**86.24**	**85.55**	**85.61**
	PCA	82.17	83.36	82.17	82.60
	AE	84.91	86.00	84.91	85.4
CIC-IDS2017	RFP	**99.70**	**99.68**	**99.70**	**99.69**
	PCA	97.77	97.94	97.77	97.77
	AE	98.54	98.65	98.54	98.56

**Table 9:** Comparison of different sampling methods.

Data set	Sampling method	Evaluation indicators (%)			
		Accuracy	Precision	Recall	F1-score
NSL-KDD	Random oversampling	98.21	98.24	98.21	98.16
	Random undersampling	87.96	93.97	87.96	89.52
	SMOTE	98.77	98.85	98.77	98.81
	ADASYN	98.73	98.82	98.73	98.75
	ENN	97.62	98.13	97.62	97.87
	RENN	98.75	98.80	98.75	98.76
	ADRDB	**99.81**	**99.76**	**99.81**	**99.79**
UNSW_NB15	Random oversampling	73.87	84.94	73.87	77.08
	Random undersampling	61.12	73.66	61.12	63.00
	SMOTE	79.38	79.61	79.38	79.50
	ADASYN	80.74	80.14	80.74	80.14
	ENN	78.05	83.72	78.05	80.01
	RENN	78.69	83.31	78.69	79.22
	ADRDB	**85.55**	**86.24**	**85.55**	**85.61**
CIC-IDS2017	Random oversampling	92.13	91.87	92.13	89.82
	Random undersampling	34.72	90.95	34.72	39.76
	SMOTE	94.68	9.85	94.68	94.33
	ADASYN	95.58	94.88	95.58	95.02
	ENN	96.63	97.01	96.63	96.65
	RENN	97.31	97.51	97.31	97.41
	ADRDB	**99.70**	**99.68**	**99.70**	**99.69**

**Table 10:** Comparison of different pooling methods.

Data set	Pooling method	Evaluation indicators (%)			
		Accuracy	Precision	Recall	F1-score
NSL-KDD	Average pooling	98.96	98.95	98.96	98.94
	Max pooling	98.42	98.38	98.42	98.39
	Average pooling + max pooling	**99.81**	**99.76**	**99.81**	**99.79**
UNSW_NB15	Average pooling	85.21	85.53	85.21	85.30
	Max pooling	84.46	85.68	84.46	85.07
	Average pooling + max pooling	**85.55**	**86.24**	**85.55**	**85.61**
CIC-IDS2017	Average pooling	98.55	98.64	98.55	
	Max pooling	93.85	94.52	93.85	93.89
	Average pooling + Max pooling	**99.70**	**99.68**	**99.70**	**99.69**

**Table 11:** Comparison of different models.

Data set	Algorithm	Evaluation indicators (%)			
		Accuracy	Precision	Recall	F1-score
NSL-KDD	Random forest	75.41	84.00	75.41	77.53
	K-means clustering	79.34	78.01	79.34	76.28
	Decision tree	76.92	71.98	54.52	55.97
	S-ResNet [49]	98.33	98.39	98.33	98.34
	CNN [50]	97.78	97.74	97.78	97.75
	CNN-GRU [51]	99.15	99.15	99.15	99.15
	CNN-LSTM [21]	98.64	98.61	98.64	98.56
	CNN-BiLSTM [52]	99.22	99.18	99.14	99.15
	SRFCNN-BiGRU	**99.81**	**99.76**	**99.81**	**99.79**
UNSW_NB15	Random forest	75.41	84.00	75.41	77.53
	K-means clustering	70.93	82.42	70.91	76.23
	Decision tree	73.37	80.94	73.36	76.30
	S-ResNet [49]	83.8	85.0	83.8	84.4
	CNN [50]	82.9	82.6	82.9	82.7
	CNN-GRU [51]	84.3	83.7	84.3	84.0
	CNN-LSTM [21]	82.6	81.9	82.6	80.6
	CNN-BiLSTM [52]	82.08	82.68	80.00	81.32
	SRFCNN-BiGRU	**85.55**	**86.24**	**85.55**	**85.61**
CIC-IDS2017	Random forest	98.21	98.58	93.40	95.92
	K-means clustering	95.03	96.40	95.21	95.80
	Decision tree	96.60	97.62	96.66	97.14
	S-ResNet [49]	95.94	96.10	95.94	95.41
	CNN [50]	89.14	84.18	89.14	85.56
	CNN-GRU [51]	99.42	99.34	99.42	99.38
	CNN-LSTM [21]	96.64	96.87	96.64	96.45
	CNN-BiLSTM [52]	99.43	99.39	99.42	99.40
	SRFCNN-BiGRU	**99.70**	**99.68**	**99.70**	**99.69**

## Data Availability

All data used in this paper can be obtained by contacting the authors of this study.
